# Presentation of the Subject of State Control of Tuberculosis1Read before the Thirty-fourth Annual Meeting of the United States Veterinary Medical Association. Nashville, Tenn., September, 1897.

**Published:** 1897-10

**Authors:** John M. Parker

**Affiliations:** Member of the Massachusetts Cattle Commission, Haverhill, Mass.


### THE JOURNAL
OF
### COMPARATIVE MEDICINE AND VETERINARY ARCHIVES.

Vol. XVIII. *OCTOBER, 1897.* No. 10.

### PRESENTATION OF THE SUBJECT OF STATE CONTROL OF TUBERCULOSIS.[1]

By John M. Parker, D.V.S.,
MEMBER OF THE MASSACHUSETTS CATTLE COMMISSION, HAVERHILL, MASS.

Before considering more fully the subject-matter of this paper, you will pardon me if I stop for a moment to notice one or two stray thoughts that have occasionally occurred to me.

It has seemed sometimes, in discussing this subject, that there has been too great a tendency among the members of the profession to look upon everything as secondary to the question whether a cow reacted to tuberculin or not. It has seemed as if there had been too great a tendency to let everything else go, to pay no attention to anything except tuberculin and tuberculosis. I have often felt that this whole matter should be taken up in a broader, more general way; that the important question of wholesome milk should receive greater attention than heretofore. The work being done by sanitary boards should be broadened and extended. They should pay greater attention to the value of cleanliness and light, ventilation, and drainage. Tuberculosis is not the only evil to be avoided, and in attending to the surroundings all are helped, and both consumer and producer are benefited.

Again, it has seemed to me, sometimes, as if there had been too much anxiety among certain veterinarians to increase and exaggerate such danger as there might be, not so much for the good of the public as because the more the people become panic-stricken, the greater the likelihood that their pocket-books would be fattened.

On the other hand, it is only since the introduction of tuberculin

[1] Read before the Thirty-fourth Annual Meeting of the United States Veterinary Medical Association, Nashville, Tenn., September, 1897.

that we have had any reason to hope that we may finally be able to control the spread of tuberculosis among dairy-cattle. Tuberculin has not only enabled us to rid many individual herds, at least temporarily, of tuberculosis, but it has greatly modified and changed the generally accepted opinion of tuberculosis. It has enabled us to gain a truer insight into the mysteries of heredity, and it has shown more clearly the prevalence of tuberculosis.

Probably one of the most important of the new facts brought to our notice through the use of tuberculin is a knowledge of the immense number of latent or undeveloped cases of tuberculosis that exist in every herd. It is startling to see a large number of autopsies made, and note what a large proportion show only very slight evidence of disease.

In referring to this matter, Bang says: "As soon as tuberculin-inoculation was undertaken on a large scale, the public was surprised at the very large number of reacting animals, even in herds of healthy appearance. I refer to the work done first by Koepp in Dorpat, then by Siedamgrotzky and Eber in Saxony, Nocard in France, Malm in Norway, and others. The Danish investigations have yielded exactly similar results. Where tuberculosis exists it often has an extent which no one had suspected. The discovery produces at first a benumbing effect, and is always very painful to the owner of dairy-stock. Because of this it cannot be made too emphatic that there is a great difference between what was formerly termed tuberculosis and what, as a result of the tuberculin-test, is now indicated by the same word. When a reacting animal is butchered, very often the butcher finds no trace of tuberculosis, and the veterinarian must search carefully to discover the little knots on the lymph-glands, frequently the only pathological signs. These are seen especially in the retro-pharyngeal, mesenteric, mediastinal and bronchial glands. The majority of reacting cows have simply latent tuberculosis. In time this may develop further in many cases. But my investigations have shown that such tuberculosis can often remain without development for years and exert no influence on the general health or the functions of the animal. We cannot conclude from this observation that an animal which reacts with tuberculin is thereby condemned to advancing disease, to wasting away, and, finally, death. The reaction simply indicates the possibility of such a result. Whether or not it will prove a reality, we do not know."

Prof. Theobald Smith, in an address to the Harvard Medical Alumni, notices the same thing when he says: "The need of a better education in animal pathology is well shown by the work undertaken in this country, by public authority, to restrict, suppress, or eradicate tuberculosis among cattle. Tuberculin produces a temporary fever in all infected animals, whether they be in the earliest stage of the disease or in the most advanced stages. In fact, the not infrequent absence of any lesion after a tuberculin reaction is explained by the statement that the focus or foci could not be detected. There are a large number of cattle slaughtered on the evidence furnished by tuberculin which are in the earliest stages of the disease. A small focus in a mediastinal gland, perhaps, or in one of the throat or mesenteric glands, is all that can be found. Pathology has informed us that traces of stationary or healed tuberculosis, unrecognized during life, are not infrequently encountered on the post-mortem table. Are such persons to be placed within the class of diseased or simply infected? I believe that an infected animal is not necessarily an affected or diseased animal, and that no one can predict just what is going to become of a primary focus, whether it will gradually encroach upon neighboring organs or become generalized, or become calcified or cicatrized." (*Boston Medical and Surgical Journal*, September 24, 1896, p. 320.)

Practically the same thing was demonstrated by Pizzini, who has reported "observations upon lymph-glands of persons having died without showing any evidence of tuberculosis. In twelve out of thirty cases examined microscopically and by inoculation in guinea-pigs, tuberculosis was demonstrated and the further fact shown that only those guinea-pigs inoculated with bronchial glands showed any evidence of disease." (*Annual of Medical Sciences.*)

This is further corroborated by reported observations made at the Johns Hopkins University and elsewhere, from the large number of autopsies made on people dying from causes other than consumption, that show evidence of undiscovered or healed tuberculosis. From the number of cases in which this occurs it is evident that a larger number of people have consumption at one time or another during life, and recover, than there are that die from this disease.

Such a state of affairs is significant, and the only inference to be drawn is that tuberculosis is far more prevalent among human beings than we have had any idea of, and, in fact, that practically the same conditions exist among people that have been found, through the use of tuberculin, to exist in cattle; and if tuberculin were used as a diagnostic agent among people we would probably find that in a large number of cases individuals would react to the test that are at present considered healthy. Under these circumstances we would repeat the question asked by Prof. Smith: "Shall all these people be considered diseased or only infected?" We incline to the belief that there is a considerable difference between such *infected* people and those that are *affected* or *diseased*. In this connection it may be of interest to glance for a moment at the statistics showing the reduction in the death-rate from tuberculosis in Massachusetts. During the past forty-five years consumption has steadily and uniformly decreased; the maximum and minimum death-rates being 42.7 per ten thousand in 1853, and 22.7 in 1893. In Glasgow, Scotland, the figures are almost exactly similar. In the period from 1860 to 1864 phthisis reached its highest mortality, viz., 4094 per million; in the period from 1890 to 1894 it was 2315, so that in twenty-five years consumption has been reduced 44 per cent. in fatality without special treatment as an infectious disease. Unfortunately, phthisis is the only form of tuberculosis which has been throughout consistently classified by the Registrar-general as a cause of death. Then the report goes on to say: "We are, therefore, shut up to the twelve years, 1883 to 1894, for evidence of the movement of tuberculous diseases *other than phthisis*. Dividing them into two periods of six years, we find that the death-rate from 'phthisis' has fallen from 2849 per million to 2316, and from 'other tubercular diseases,' from 1090 per million to 884—in both cases 19 per cent., a result which quite casts into the shade the improvement in Prussia and Saxony, quoted from Cornet, which he puts down to the credit of special prophylaxis. Clearly, then, we are warranted in asserting that among infectious diseases tuberculosis is the most amenable of all to general hygienic measures; that, in fact, from these alone as good results are obtained as from hygienic measures plus isolation, disinfection, etc., in the case of diseases popularly known as infectious. It is not implied that special measures directed against the infectivity might not have produced even better results; but in view of the difficulties in the way of special prophylaxis it is to be contended that more is to be expected from hygiene." (*Report Glasgow Board of Health.*)

The special interest attaching to the reduced mortality from this disease in both Massachusetts and Glasgow seems to me to be due to the fact that these improved conditions have been attained entirely as a result of a better knowledge of the disease and a more strict carrying out of hygienic measures. Such a retrospect is encouraging when one considers that similar results would follow the introduction of like measures when applied to dairy-cattle; but before this can be done people must recognize the necessity for it and the great room for improvement there really is.

Before considering in greater detail the control work that has been done in Massachusetts, let us turn for a moment to some of the other countries and States where such work has been attempted. In European countries, probably the laws in force in Denmark are best known. There for many years past owners have attempted to control tuberculosis by removing diseased animals and having the stalls disinfected, and, as Bang remarks: "These efforts were not without significance. The conditions of health were improved, especially in places where the entire herd was subjected to thorough investigation by a competent veterinarian, and where the owner and veterinarian worked harmoniously together. . . . But even with these conditions it was hardly possible to be entirely freed from the disease. . . . The hidden, latent cases always remained, and were continuously coming to the surface." After the introduction of tuberculin Bang conceived the idea of dividing the reacting animals from those that did not react, and when the reacting animals showed no physical evidence of disease they were used for breeding purposes, and their progeny immediately separated and kept with the healthy lot. In this way a new, healthy herd was gradually built up. The State assisted by providing tuberculin and veterinary services free, and animals showing physical evidence of disease are destroyed. In Switzerland a somewhat similar plan has been adopted; there the State pays half the cost of test, and only entire herds can be tested. All cattle that react must be branded. The French method is slightly different; there, if an animal is found to be diseased, the State tests the entire herd, the owner having the option of keeping those that react for one year or slaughtering them immediately and receiving compensation from the State. All cattle coming into the country must also be tested. The government of the Dominion of Canada provides tuberculin free, this being done principally for educational purposes. It is also proposed to test herds in which disease is known to exist, and, as in France, all cattle coming into the country must be tested. In Ontario the Board of Agriculture has strongly advocated Bang's method among the farmers. If the milk, they say, from the reacting cows is used for the calves it should be Pasteurized. They state that "it is advisable to fatten quickly the animals that give a reaction with tuberculin, but are apparently healthy, and sell them to the butcher, making calculations to get rid of all reacting animals in the course of eighteen months or two years." They also state "up to the present time these tests for tuberculosis by means of tuberculin have been carried on by experts, veterinarians, etc., but that is no reason why an intelligent man who makes an effort to post himself as to the methods of keeping clean and using the various instruments should not test his own herd from time to time." In New York, New Jersey, New Hampshire, Maine, Vermont, and Rhode Island, owners receive half valuation for cattle killed and found diseased, and full valuation if condemned and killed and found free from disease. Pennsylvania, Connecticut, Illinois, Indiana, and other States give limited appraised value. In most of these States tuberculin is used either at the owner's request or in such herds as are known to be diseased. Connecticut relies almost entirely on physical examination. In Pennsylvania, New Hampshire, and Massachusetts experimental work is being conducted in various directions at the present time, and the results will be well worth noting. In Massachusetts the principles are practically the same as in these other States. The State pays full value for diseased animals, with a limit of sixty dollars. All cattle coming into the State must be tested, and tuberculin may also be used as an aid to diagnosis on animals condemned on physical examination by a qualified veterinarian and at the written request of owners, provided they agree in their turn to certain conditions prescribed by the Cattle Commissioners.

In Massachusetts the work of inspection has been, I think, fairly successful. In considering it I would divide it into two divisions: First, as a factor in the protection of the public health; second, as a help to the farmers and stock-owners in their endeavors to rid their herds of disease. It seems to me that these two points must be considered separately. Many facts must be considered, and the matter must be looked at from many points of view before one can gain even a faint idea of the magnitude of the subject, and it seems to me that in view of the many interests involved, since we cannot stamp it out by heroic measures, we should be content if we can make steady and substantial gain. We have only to look back a very few years to the time when milk was sold and cattle were butchered without any kind of supervision whatsoever. At that time the meat from "coughers" and "bolognas" was placed on the market, and milk was sold without any restriction so far as the health of the cow was concerned.

In 1876 (according to the *Report of the Cattle Commissioners for*

1895) an act was passed providing for the appointment of inspectors of provisions and animals intended for slaughter. This was a permissive act, simply providing that the mayor and aldermen of cities and the selectmen of towns may annually appoint one or more persons who may inspect all provisions and animals intended for slaughter. Under this act, which was taken advantage of by the cities and towns only slightly, it was found to be impossible to organize any systematic inspection of the herds of cattle in the State, and in 1892 the law was so amended as to provide that the same authorities "shall annually in the month of April appoint one or more persons to be inspectors of provisions and of animals intended for slaughter or kept for the production of milk." Under this latter act, however, no penalty was provided for the failure of the cities and towns to make the appointments required by the act, and it was again found necessary to strengthen the law. Accordingly, in 1893, a penalty was provided for cities and towns failing to appoint inspectors as required by law, and, further, the inspectors were, by this act, and for the first time, brought under the partial control of the Cattle Commissioners, by giving the Board the right to make appointments where cities or towns failed to do so, and the power to remove incompetent inspectors. This latter act was approved May 3, 1893, and immediately after its passage this Board undertook to collect the names and addresses of the various inspectors; to instruct them in their duties in so far as they related to the matter of the suppression of contagious diseases among the domestic animals; and to see that, so far as possible, animals intended for slaughter or kept for the production of milk were inspected as thoroughly as circumstances would permit. The first attempt to collect the names and addresses of the various inspectors was begun on May 12, 1893. Up to about October 15, 1893, only about one-half of the cities and towns in the Commonweatlh had complied with this requirement. From this small beginning and in the face of many discouragements, the organization of this corps of inspectors has been developed until now (1895) all but four of the cities and towns in the Commonwealth have made the necessary appointments."

This was a long step in the right direction. The principle involved was right. It secured the inspection of each herd in the State, and, as I shall endeavor to show later, it has resulted in an immense amount of good. Of course, the system is by no means perfect, the local authorities, except in large towns, rarely appoint qualified men for the position. I hope, however, that before long, instead of the local town inspector, the State will be divided into districts, each district being in charge of an experienced, qualified veterinarian. In this way, the work will be done far more thoroughly, and one of the principal objections to the present method will be done away with.

In referring to the work of the local inspectors one often hears the remark made, that physical examination does not amount to much any way. Now, while I admit that many bad cases cannot be picked out by physical examination, yet I am also satisfied that a large proportion of the bad cases of tuberculosis can be picked out, and many more can be suspected, so that they may be quarantined and tested. As a rule, the bulk of cases of udder-tuberculosis and a large proportion of generalized tuberculosis will usually show some suspicious symptom, and if these bad cases are got rid of, and if the milk supply is obtained from such animals as are in good general health and show no physical evidence of disease, I believe the danger would be reduced to a minimum.

As showing the good that can be accomplished by a herd-to-herd inspection, even by those who cannot be considered experts, I wish to call your attention to the following tables; this refers to the work done by the local inspectors for the past three years, and while the tables are not to be considered absolutely correct, especially for the year 1895, yet I believe they give in a fairly accurate way, at least, the approximate number of bad, dangerous cases that have occurred in each year.

1895, January 1st to December 31st: Animals tested, 4484; found diseased and condemned, 2398, or 53.4 per cent.; general tuberculosis, 784, or 32.6 per cent. Animals tested, no reaction, permission to kill; post-mortem lesions found, 53.

1896, January 1st to December 31st: Number of animals tested, 7062; number of animals found diseased and condemned, 4173, or 59 per cent.; general tuberculosis, 1051, or 25.1 per cent. Animals tested, no reaction, permission to kill; post-mortem lesions found, 82.

1897, January 1st to June 30th: Number of animals tested, 5300; number of animals found diseased and condemned, 3016, or 56.9 per cent.; general tuberculosis, 84, or 2.7 per cent. Animals tested, no reaction, permission to kill; post-mortem lesions found, 21.

You will notice, of course, that the last year is incomplete; the table only takes up to the end of June, but in the time it includes the yearly inspection, which was carried on this year in the spring instead of the fall, and at the present time the great bulk of the work is over and only a very few cattle are being placed in quarantine by the local inspectors.

Further, there were more animals quarantined this year than in 1895. In 1895, 4484 animals were quarantined, and 53.4 per cent. were condemned and found diseased, and 32.6 per cent. had generalized tuberculosis. In 1896, 7062 animals were quarantined, and 59 per cent. were found diseased and condemned, and 25.1 per cent. had generalized tuberculosis, an increase in the number of cases of tuberculosis and a considerable decrease in the cases of generalized tuberculosis from the previous year. In 1897, 5300 were quarantined, and 56.9 per cent. condemned because of reaction, and found diseased, and only 2.7 per cent. had generalized tuberculosis. This means simply that the bad, dangerous cases have been got rid of to a great extent, and I believe by perfecting this system the conditions can be still further improved, and all danger to the public health from this source practically eliminated. It is, I know, an immense improvement over previously existing conditions.

[To be continued.]

